# Personalized Cardiac Rehabilitation: Integrating Biomarker‐Guided, Technology‐Enabled, and Population‐Specific Strategies in Exercise Medicine

**DOI:** 10.1155/crp/2912143

**Published:** 2026-10-27

**Authors:** Tong Liu, Yuting Xia, Li Wei

**Affiliations:** ^1^ Chengdu Integrated TCM&Western Medicine Hospital Chengdu 610095 Sichuan China; ^2^ Department of Pediatrics Key Laboratory of Birth Defects and Related Diseases of Women and Children of MOE West China Second University Hospital Sichuan University Chengdu 610041 Sichuan China scu.edu.cn

**Keywords:** artificial intelligence, cardiac rehabilitation, exercise medicine, individualized therapy, population specificity, precision medicine, wearable technology

## Abstract

Cardiovascular diseases remain the leading global cause of mortality and morbidity, necessitating continuous advancement in secondary prevention strategies. Cardiac rehabilitation (CR), a cornerstone of comprehensive cardiovascular care, has been significantly enhanced by innovations in exercise medicine, evolving towards more personalized and accessible paradigms. This review synthesizes the current frontiers in exercise‐based CR, focusing on the synergistic integration of three transformative domains: technological innovation, precision medicine, and population‐specific strategies. We first delineate the established physiological benefits and evidence‐based efficacy of exercise therapy within CR frameworks. Subsequently, we examine the application of digital and intelligent technologies—including wearable devices, remote monitoring, artificial intelligence (AI), and big data analytics—which enable dynamic, data‐driven patient management and remote care delivery. The paper further explores the paradigm of precision exercise prescription, guided by biomarkers, genetic profiling, and other individual biological data, to tailor interventions for optimal safety and therapeutic yield. Special emphasis is placed on addressing the heterogeneous needs of key patient populations, such as the elderly, women, and those with specific comorbidities like heart failure or arrhythmias, by outlining individualized strategies that improve engagement and effectiveness. Finally, we discuss future directions, highlighting multimodal technology platforms and in‐depth precision medicine applications as key drivers for the next generation of CR, while critically evaluating the evidence readiness of emerging technologies and addressing implementation barriers. This review aims to inform clinical practice and guide future research towards developing more personalized, technology‐enabled, and equitable cardiac rehabilitation models.

## 1. Introduction

Cardiovascular diseases (CVD) remain the leading cause of morbidity and mortality worldwide, representing a significant public health challenge that necessitates comprehensive management strategies. The burden of CVD is particularly pronounced in the aging population, where conditions such as coronary artery disease (CAD), heart failure (HF), and peripheral artery disease are prevalent. As the global population ages, the demand for effective interventions to improve outcomes in patients with cardiovascular conditions has become increasingly urgent. Cardiac rehabilitation (CR) has emerged as a cornerstone in the secondary prevention of CVD, demonstrating substantial benefits in enhancing patients’ functional capacity, quality of life, and overall prognosis. Indeed, CR encompasses a holistic approach that integrates exercise training, dietary modifications, psychosocial support, and comprehensive management of cardiovascular risk factors, aiming to optimize recovery and prevent further complications [1].

This is a narrative review that synthesizes current evidence and emerging trends in exercise‐based CR. A comprehensive literature search was conducted in PubMed, Web of Science, and Scopus for articles published between January 2015 and June 2026, using combinations of keywords including “cardiac rehabilitation,” “exercise medicine,” “wearable technology,” “artificial intelligence,” “precision medicine,” “biomarkers,” and “individualized therapy.” Additional relevant studies were identified through manual screening of reference lists of included articles and key reviews. Priority was given to high‐quality meta‐analyses, systematic reviews, and randomized controlled trials, while early‐phase and proof‐of‐concept studies were included to illustrate emerging directions.

To provide readers with a clear framework for evaluating the strength of evidence underlying the technologies and approaches discussed throughout this review, we adopt a four‐tier evidence hierarchy: Tier I—established clinical practice supported by multiple randomized controlled trials and meta‐analyses; Tier II—promising interventions supported by moderate‐quality evidence including pilot RCTs and large observational studies, warranting further investigation; Tier III—emerging approaches supported by proof‐of‐concept studies, feasibility trials, or early‐phase investigations, requiring validation before clinical adoption; and Tier IV—speculative or theoretical applications lacking direct CR‐specific evidence, presented to illustrate future directions. Throughout this review, we explicitly classify the evidence level for each major technology and approach to guide clinical interpretation and prioritization. A comprehensive summary of the evidence readiness and clinical adoption potential of all technologies discussed in this review is provided in Table 1.

**TABLE 1:** Evidence readiness and clinical adoption potential of technologies in cardiac rehabilitation.

Technology	Current evidence level (Tier I–IV)	Key evidence gaps	Readiness for clinical adoption
Wearable devices (HR/activity monitoring)	Tier II	Validation across diverse populations; accuracy during high‐intensity exercise; long‐term adherence data	Ready for implementation with caveats
Remote monitoring platforms	Tier II	Long‐term outcomes; cost‐effectiveness	Ready for implementation with caveats
AI/ML for exercise prescription	Tier III	Large RCTs; prospective validation; integration with clinical workflows	Pilot/feasibility phase
Computer vision for posture correction	Tier IV	CR‐specific validation; user acceptance studies	Research phase
NLP/chatbots for patient engagement	Tier III	CR‐specific RCTs; clinical outcome data	Pilot/feasibility phase
Biomarker‐guided protocols (troponin, hs‐CRP, NT‐proBNP)	Tier III	Standardized thresholds; cost‐effectiveness; prospective trials	Research phase
Circulating miRNA biomarkers	Tier IV	Clinical validation; assay standardization; CR‐specific studies	Research/translational phase
Genetic testing for exercise response	Tier III	Prospective validation; population‐specificity; clinical utility studies	Research phase
VR/XR for exercise engagement	Tier IV	CR‐specific RCTs; elderly usability; long‐term outcomes	Speculative
Digital twins	Tier IV	CR‐specific evidence; validation of predictive accuracy	Speculative
Blockchain for data security	Tier IV	CR‐specific evidence; regulatory frameworks; implementation studies	Speculative
Advanced omics/microbiome	Tier IV	CR‐specific evidence; clinical interpretation; cost‐effectiveness	Speculative

*Note:* Tier I = established practice (multiple RCTs/meta‐analyses); Tier II = moderate evidence (pilot RCTs/large observational studies); Tier III = emerging (proof‐of‐concept/feasibility studies); Tier IV = speculative/theoretical (no direct CR‐specific clinical data).

Traditional models of CR, however, face several challenges, including low patient adherence and a lack of personalized approaches tailored to individual needs. Many patients, particularly the elderly and those with comorbidities, are often underrepresented in CR programs, leading to suboptimal outcomes. Studies have shown that while CR can significantly improve exercise capacity and quality of life, barriers such as kinesiophobia, anxiety, and depression can impede participation and engagement in these programs. Addressing these psychosocial factors is crucial for enhancing adherence and maximizing the benefits of rehabilitation [2]. Furthermore, the integration of technology into CR, such as mobile health (mHealth) applications and tele‐rehabilitation, has shown promise in overcoming geographical and logistical barriers, thereby increasing accessibility and flexibility for patients [3].

The rapid advancements in exercise medicine and technology present new opportunities for CR. Innovations in personalized medicine, which emphasize the tailoring of treatment strategies based on individual patient characteristics, are becoming increasingly relevant in the field of CR. Precision medicine approaches can enhance the effectiveness of rehabilitation by considering factors such as age, gender, comorbidities, and specific cardiovascular conditions. For instance, recent studies have highlighted the effectiveness of graded CR care in improving clinical outcomes and quality of life in patients with severe coronary heart disease, indicating that tailored rehabilitation strategies can yield superior results compared to conventional methods. Additionally, the incorporation of psychosocial support and lifestyle modifications into CR programs can further enhance patient engagement and adherence, ultimately leading to improved health outcomes.

The future of CR lies in the integration of technology, precision medicine, and population‐specific strategies. By leveraging advancements in telemedicine and mHealth technologies, healthcare providers can create more accessible and personalized rehabilitation programs that cater to the unique needs of diverse patient populations. Furthermore, ongoing research into the psychosocial aspects of CR underscores the importance of addressing mental health and emotional well‐being as integral components of effective rehabilitation strategies. This comprehensive approach not only aims to improve physical health but also enhances the overall quality of life for patients undergoing CR, ultimately contributing to better long‐term outcomes and reduced healthcare costs associated with CVD [4].

The conceptual framework for this evolving paradigm is illustrated in Figure 1, which highlights the synergistic integration of three core pillars: technological innovation, precision medicine, and population‐specific strategies, all converging to enable personalized, accessible, and effective CR.

**FIGURE 1:**
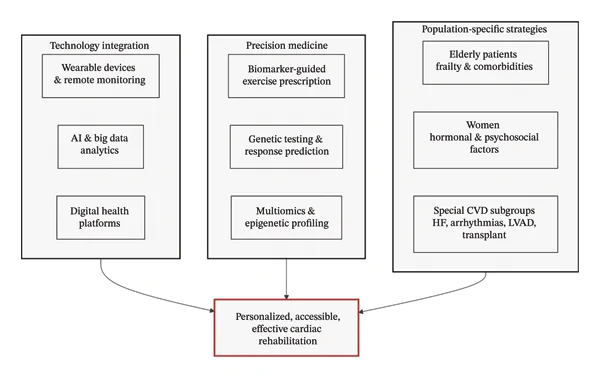
The three pillars of modern cardiac rehabilitation exercise medicine.

As the field of CR continues to evolve, it is imperative to adopt innovative solutions that address the existing challenges of low adherence and lack of personalization. The integration of technological advancements, precision medicine, and a focus on psychosocial well‐being will play a pivotal role in optimizing rehabilitation outcomes for patients with CVD. This review aims to systematically explore the latest developments in the field of CR, highlighting the potential for enhanced patient care through the adoption of innovative, individualized strategies that align with contemporary healthcare needs.

## 2. The Theoretical Basis and Clinical Value of CR Exercise Therapy

### 2.1. Physiological Effects of Exercise on the Cardiovascular System

Aerobic exercise and resistance training confer cardiovascular protection through multifaceted mechanisms: improved endothelial function, reduced inflammation, and enhanced autonomic balance. Aerobic exercise increases nitric oxide bioavailability, improving vascular compliance and reducing arterial stiffness, while lowering inflammatory markers such as interleukin‐6 and C‐reactive protein. Resistance training improves muscle mass and metabolic health. Exercise enhances parasympathetic tone and reduces sympathetic overactivity, maintaining heart rate variability (HRV) and blood pressure stability. Clinical studies confirm mixed‐type training improves cardiac autonomic function, evidenced by increased HRV and peak oxygen uptake (VO_2_peak) [5]. Exercise also promotes coronary collateral circulation via shear stress‐induced vascular remodeling and VEGF upregulation. At the cellular level, exercise induces mitochondrial biogenesis and autophagy in cardiomyocytes, enhancing energy metabolism and ischemic resistance [6, 7]. Neurohumoral adaptations include baroreflex sensitivity improvement and renin‐angiotensin‐aldosterone system modulation [8, 9]. Sex‐specific differences exist: males show greater left ventricular mass increases with resistance training, while females exhibit better diastolic function with endurance training [10]. Evening exercise may offer superior autonomic and vascular benefits [11]. These pathways are summarized in Figure 2.

**FIGURE 2:**
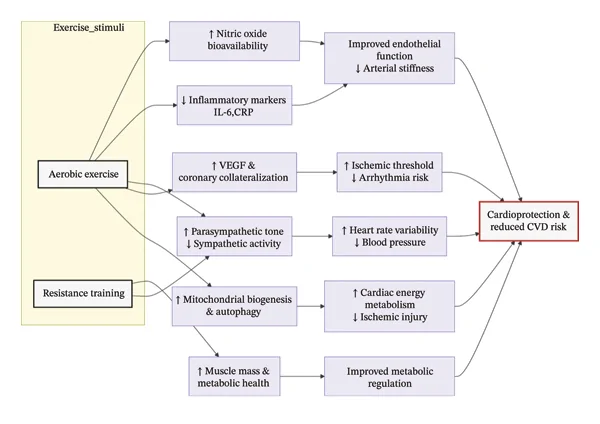
The physiological pathway of exercise‐induced cardioprotection.

### 2.2. Evidence‐Based Medical Evidence of CR

Large‐scale meta‐analyses robustly support CR efficacy in reducing cardiovascular mortality and hospital readmission rates. The updated CR outcome study (CROS II), which synthesized data from 31 studies encompassing over 228,000 patients with CAD, demonstrated that participation in CR is associated with a 20%–30% reduction in total mortality and a 25% decrease in rehospitalization rates after acute coronary syndrome (ACS) and coronary artery bypass grafting (CABG) [12]. This landmark meta‐analysis underscores the clinical effectiveness of contemporary CR programs delivered according to evidence‐based standards, although it also highlights the heterogeneity in CR delivery and the urgent need for internationally accepted minimum standards.

Exercise‐based CR, as the cornerstone of rehabilitation, significantly enhances patients’ exercise tolerance, quality of life, and psychological well‐being. Multiple randomized controlled trials and observational studies have confirmed that structured exercise training improves VO_2_peak, 6 min walk test (6MWT) distances, and muscle strength across diverse cardiac populations, including HF, post‐myocardial infarction (MI), and post‐CABG patients [13–15]. For instance, a meta‐analysis including 131 trials with nearly 10,000 HF patients revealed that exercise training leads to a clinically meaningful improvement in VO_2_peak and quality of life, while reducing hospitalizations [14]. Similarly, in patients with ACS, exercise‐based CR has been shown to improve functional capacity and reduce adverse cardiovascular events [16]. Importantly, early initiation of exercise rehabilitation, even as soon as 2 weeks post‐sternotomy, has been demonstrated to be safe and effective in improving exercise tolerance without increasing adverse events [17].

Beyond physical benefits, CR exerts favorable effects on psychological health. Psychosocial factors such as depression, anxiety, and stress are well‐recognized contributors to cardiovascular morbidity and mortality. Evidence indicates that CR programs incorporating psychological support and mind‐body interventions can alleviate these psychosocial risks, thereby improving patient‐reported outcomes and adherence to therapy [2, 18]. Systematic reviews emphasize the importance of integrating psychosocial screening and tailored interventions within CR to optimize holistic patient care [2]. Furthermore, behavioral medicine interventions embedded in physiotherapy have shown promise in enhancing psychological outcomes and health‐related quality of life, although further research is warranted to delineate their incremental benefits over routine care [19].

Emerging research also validates the role of early exercise interventions in modulating pathophysiological processes post‐ACS. Exercise‐based CR initiated early after acute coronary events can attenuate adverse left ventricular remodeling, a key determinant of HF progression, as evidenced by improvements in echocardiographic parameters and inflammatory markers [20, 21]. Additionally, early exercise rehabilitation in acute decompensated HF patients has been associated with enhanced exercise capacity and reduced rehospitalization rates [22].

Innovations in CR delivery, including home‐based and digital health‐supported models, have demonstrated comparable effectiveness to traditional center‐based programs in improving functional outcomes and adherence, particularly in older adults and patients undergoing transcatheter aortic valve replacement (TAVR) [23–25]. These approaches address barriers to participation and facilitate continuous monitoring, thereby extending the reach and impact of CR.

Evidence robustly shows exercise‐based CR reduces cardiovascular mortality and rehospitalization, improves exercise tolerance, enhances quality of life, and mitigates psychological distress. A synthesis of key outcomes from major meta‐analyses supporting the efficacy of CR across different cardiac populations is presented in Table 2. Early initiation of CR post‐cardiac events further augments these benefits, while novel delivery modalities promise to increase accessibility and adherence. Continued efforts to standardize CR protocols and integrate comprehensive psychosocial care are essential to maximize patient outcomes.

**TABLE 2:** Evidence‐based benefits of exercise‐based cardiac rehabilitation: Key outcomes from meta‐analyses.

Patient population	Primary outcome measure	Key finding	Reference
Coronary artery disease (CAD) patients post‐ACS/CABG	All‐cause mortality	∼20–30% reduction	Gore et al. (CROS‐II) [13]
Coronary artery disease (CAD) patients post‐ACS/CABG	Rehospitalization rate	∼25% reduction	Gore et al. (CROS‐II) [13]
Heart failure (HF) patients	Peak oxygen uptake (VO_2_peak)	Significant improvement	Campos et al. [15]
Heart failure (HF) patients	Quality of life	Clinically meaningful improvement	Campos et al. [15]
Heart failure (HF) patients	Hospitalization rate	Reduction	Campos et al. [15]
Post‐myocardial infarction (MI) patients	Exercise tolerance (e.g., 6MWT)	Significant improvement	Shokri et al. [16]
Acute coronary syndrome (ACS) patients	Functional capacity	Improvement	Ennis et al. [17]
Acute coronary syndrome (ACS) patients	Adverse cardiovascular events	Reduction	Ennis et al. [17]
Post‐sternotomy patients (early initiation)	Exercise tolerance	Safe and effective improvement	Pieczynski et al. (SCAR trial) [18]
Patients with CVD (general)	Psychological distress (e.g., depression, anxiety)	Mitigation through integrated psychosocial support	Kupper et al. [2]

## 3. Technology Integration: The Application of Digitalization and Intelligentization in CR

Digital tools—wearables, remote monitoring, AI, and big data—create a cohesive ecosystem for remote and personalized CR. This section reviews the current applications and evidence for these technologies, with Figure 3 providing a visual overview of the digital rehabilitation ecosystem and the flow from patient‐generated data through analytics to clinical decision support and patient engagement. Evidence classification: wearable devices and remote monitoring platforms are Tier II; AI/ML applications for exercise prescription are Tier III; NLP/chatbots for patient engagement are Tier III; and computer vision for posture correction is Tier IV (see Table 1 for complete summary).

**FIGURE 3:**
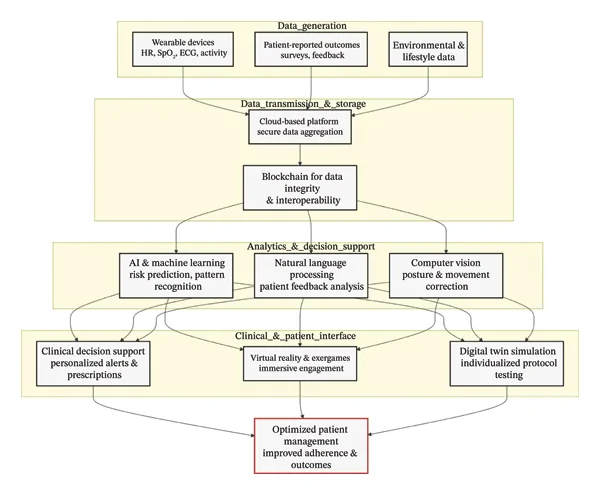
The digital ecosystem for technology‐integrated cardiac rehabilitation.

### 3.1. Wearable Devices and Remote Monitoring Technologies

Next‐generation wearable devices enable real‐time monitoring of HRV, SpO_2_, and activity intensity. Wearable ECG patches with dry electrodes detect arrhythmias and ischemic changes during exercise with high signal quality [26]. Cloud‐based platforms transmit data to clinicians for remote supervision, exercise titration, and early intervention. Clinical decision support systems (CDSS) further optimize patient management [27].

Remote CR programs achieve outcomes comparable to center‐based rehabilitation. RCTs demonstrate significant improvements in peak VO2 and anaerobic threshold in CAD patients using wearables with real‐time monitoring [28]. Online coaching further enhances physical activity without compromising safety. Smartwatch‐facilitated home‐based CR improves adherence and cardiopulmonary function and it reduces anxiety and depression [29]. Long‐term follow‐up data show sustained functional and quality‐of‐life improvements [30–36].

Wearables also support HRV biofeedback (HRVB) via IoT‐connected systems that autonomously adjust biofeedback parameters for home‐based CR [37]. Machine learning applied to wearable data can predict 6‐min walk test outcomes and guide personalized care [38].

However, challenges remain: data integration, regulatory approval, reimbursement, and accuracy across diverse populations. Caution is warranted during high‐intensity exercise, especially in HF patients, due to underestimation biases [39]. Technical issues like electrode adhesion require ongoing improvement [40]. Successful hybrid CR implementation requires system‐level resources, staff training, and patient support [41].

Adherence barriers include low self‐efficacy, fear of exercise, depression, and limited social support [42, 43]. Accessibility disparities—broadband access, device ownership, digital literacy—can exacerbate inequities if unaddressed. Hybrid models combining remote monitoring with periodic coaching are recommended.

Systematic reviews generally support wearable devices’ positive impact on physical activity and adherence, though methodological heterogeneity tempers conclusions [44]. Continued research and implementation efforts are essential (evidence tier: wearable devices and remote monitoring platforms—Tier II; see Table 1 for complete summary).

### 3.2. AI and Big Data Analytics

AI and big data analytics enable precise risk prediction, personalized exercise prescriptions, and adherence monitoring. ML algorithms analyze rehabilitation data to identify patterns and optimize regimens. In CAD patients, AI‐driven telerehabilitation improved exercise capacity and adherence. A BERT‐based NLP model analyzing patient feedback showed enhanced satisfaction and compliance [45].

Computer vision assesses and corrects exercise posture in real‐time, reducing injury risk—particularly valuable in remote settings [46, 47]. NLP‐powered conversational agents provide continuous patient support, detect adherence inconsistencies, and tailor educational content [45].

Implementation challenges for AI include (1) clinician training gaps, (2) EHR interoperability issues, (3) evolving regulatory frameworks, (4) reimbursement gaps, and (5) digital literacy disparities. Addressing these requires coordinated efforts from policymakers, institutions, developers, and professional societies.

AI also supports interpretable ML models for complex populations like cardio‐oncology patients [48]. However, most AI applications in CR are supported by proof‐of‐concept or small pilot studies; large RCTs are urgently needed.

Data quality, algorithm transparency, and clinical workflow integration require careful attention to ensure AI complements, rather than replaces, clinical judgment. Privacy and equitable access must also be addressed [47, 49] (evidence tier: AI/ML for exercise prescription—Tier III; NLP/chatbots—Tier III; computer vision—Tier IV; see Table 1 for complete summary).

## 4. Application of Precision Medicine in Exercise Prescription for CR

### 4.1. Biomarker‐Based Personalized Protocols

Biomarkers enable personalized exercise prescriptions, allowing for tailored interventions that optimize patient outcomes. One pivotal biomarker in this context is cardiac troponin, particularly its release patterns induced by exercise. Exercise‐induced elevations in muscle‐specific troponins reflect transient myocardial stress or injury and can serve as a guide to individualize exercise intensity. For instance, patients exhibiting pronounced troponin release during moderate exercise may benefit from adjusted, lower‐intensity regimens to avoid exacerbating myocardial injury, while those with minimal troponin response could safely engage in higher intensity training. This biomarker‐guided approach ensures that exercise prescriptions align with the patient’s myocardial tolerance, reducing risks and enhancing safety in rehabilitation programs [50].

Inflammatory and metabolic biomarkers also provide dynamic insights into exercise adaptation and cardiovascular health status. High‐sensitivity C‐reactive protein (hs‐CRP), a sensitive marker of systemic inflammation, has been shown to decrease following structured CR, particularly with high‐intensity aerobic interval training (HIAIT). Such reductions in hs‐CRP correlate with improved endothelial function and reduced cardiovascular risk, indicating that monitoring hs‐CRP can reflect the anti‐inflammatory benefits of exercise and guide adjustments in training intensity and duration. Similarly, metabolic markers such as N‐terminal pro‐B‐type natriuretic peptide (NT‐proBNP), which reflects myocardial wall stress, dynamically change with exercise and rehabilitation. Decreases in NT‐proBNP levels post‐intervention signify improved cardiac function and reduced HF severity, making it a valuable biomarker to monitor therapeutic efficacy and personalize exercise regimens in HF patients [50, 51].

While these biomarker‐guided approaches are biologically plausible and supported by preliminary evidence, their clinical utility remains to be validated in prospective trials. Thresholds for guiding exercise prescription have not been standardized, and the cost‐effectiveness of routine biomarker monitoring in CR has not been established.

### 4.2. Emerging Biomarkers: Circulating MicroRNAs

Emerging research highlights the promising role of circulating microRNAs (miRNAs) as novel biomarkers for predicting and monitoring exercise responses.

These small, noncoding RNAs regulate gene expression post‐transcriptionally and are released into circulation in response to physiological stress, including exercise. Specific miRNA expression profiles have been associated with cardiovascular remodeling, inflammation, and metabolic regulation. For example, exercise‐induced modulation of miRNAs involved in angiogenesis and cardiac repair pathways could serve as predictive tools for identifying patients who will benefit most from particular exercise modalities. The noninvasive nature of circulating miRNA profiling, combined with their sensitivity to subtle molecular changes, positions them as next‐generation biomarkers for precision exercise medicine. Although clinical application is still in early stages, integrating miRNA signatures into rehabilitation protocols holds the potential to refine patient stratification and optimize individualized exercise prescriptions [50].

An overview of established and emerging biomarkers, along with their utility in guiding exercise prescription, is provided in Table 3.

**TABLE 3:** Markers and parameters for precision exercise prescription in cardiac rehabilitation.

Marker/parameter category	Specific marker/parameter	Physiological/cardiac relevance	Utility in exercise prescription and monitoring
Myocardial injury/stress	Cardiac troponin (cTnI, cTnT)	Gold‐standard marker of myocardial cell injury or stress. Exercise‐induced release reflects transient myocardial demand or microinjury.	Safety and intensity guidance monitors myocardial tolerance. Patients with pronounced release during moderate exercise may require lower‐intensity regimens. Minimal response may allow safe progression to higher intensities.
Inflammation	High‐sensitivity C‐reactive protein (hs‐CRP)	Sensitive marker of systemic inflammation. Elevated levels are associated with endothelial dysfunction and increased cardiovascular risk.	Efficacy monitoring tracks anti‐inflammatory effects of exercise (esp. HIAIT). Reduction post‐CR correlates with improved endothelial function and reduced CV risk, guiding training intensity/duration.
Myocardial wall stress and HF severity	N‐terminal pro‐B‐type natriuretic peptide (NT‐proBNP)	Released in response to myocardial stretch and volume overload. Key biomarker for diagnosing and monitoring heart failure (HF) severity.	Therapeutic response and personalization in HF: decreasing levels post‐intervention signify improved cardiac function. Used to personalize and titrate exercise regimens in HF patients and monitor therapeutic efficacy.
Novel/emerging	Circulating MicroRNAs (miRNAs) e.g., angiogenic miRNAs (miR‐126)	Small noncoding RNAs regulating post‐transcriptional gene expression. Released in response to physiological stress (exercise). Involved in CV remodeling, inflammation, angiogenesis, and repair.	Future‐predictive and stratification tool: potential to predict individual response to specific exercise modalities (e.g., angiogenesis‐promoting exercise). Noninvasive profiling may refine patient stratification for ultra‐personalized Rx. (research/translational stage).
Metabolic and functional adaptation	Peak oxygen uptake (VO_2_peak)	Gold‐standard measure of cardiorespiratory fitness and integrative cardiovascular/pulmonary/muscular function.	Foundation for prescription and progress: primary objective measure for exercise prescription (intensity zones) and the key metric for tracking functional improvement throughout CR.

*Note:* This table includes both conventional molecular biomarkers (troponin, hs‐CRP, NT‐proBNP, and miRNAs) and functional parameters (VO_2_peak) that collectively inform individualized exercise prescription. While VO_2_peak is a functional test parameter rather than a molecular biomarker, it is included here as a critical clinical metric for exercise intensity titration and prognostic assessment.

Biomarker‐guided CR is evolving, leveraging exercise‐induced troponin release patterns, inflammatory and metabolic markers such as hs‐CRP and NT‐proBNP, and the emerging field of circulating miRNAs. This integrative biomarker approach facilitates precise modulation of exercise intensity, duration, and modality, enhancing safety and efficacy. Future research should focus on validating biomarker thresholds for clinical decision‐making and developing accessible assays to implement these personalized strategies broadly in CR settings (evidence tier: troponin, hs‐CRP, NT‐proBNP‐guided protocols—Tier III; circulating miRNAs—Tier IV; see Table 1 for complete summary).

### 4.3. Genetic Testing and Prediction of Exercise Response

Genetic testing has emerged as a promising tool to predict individual variability in response to exercise training, particularly in the context of CR and physical fitness improvements. Specific gene polymorphisms, such as the angiotensin‐converting enzyme (ACE) insertion/deletion (I/D) polymorphism, have been implicated in modulating the physiological adaptations to exercise. For example, ACE gene variants influence skeletal muscle hypertrophy, cardiac remodeling, erythropoiesis, endurance capacity, and susceptibility to injury, thereby affecting exercise outcomes [52]. The ACE I/D polymorphism modulates ACE activity, which in turn shapes the molecular pathways underlying exercise adaptations. Pharmacological inhibition of ACE selectively attenuates certain adaptations, such as cardiac and hematological responses, without affecting peripheral muscle adaptations or aerobic performance, highlighting the nuanced role of ACE in exercise physiology. While the ACE I/D polymorphism is among the most studied genetic variants in exercise science, its clinical utility for individualizing CR prescriptions remains uncertain. The following discussion addresses both current knowledge and future research directions.

Beyond single gene polymorphisms, genome‐wide association studies (GWAS) have identified multiple genetic loci associated with exercise adaptability. A GWAS involving 507 participants undergoing interval and continuous exercise training identified 12 novel loci suggestively associated with changes in VO_2_peak, a key measure of cardiorespiratory fitness [53]. Although no variants reached genome‐wide significance after adjustment for covariates, the study developed a polygenic predictor score (PPS) based on these loci. While the PPS did not robustly predict VO_2_peak response in validation cohorts, significant correlations of variant effects suggest that with larger sample sizes, more definitive genetic predictors may be identified. This highlights the complex polygenic nature of exercise response and the need for ongoing research to validate genomic predictors for clinical application.

It must be emphasized that genetic predictors of exercise response are still in early research phases. The clinical applicability of polygenic scores remains limited by small effect sizes, population‐specificity, and lack of prospective validation studies.

Pharmacogenomics further informs the optimization of exercise and drug therapies. For instance, in HF patients with reduced ejection fraction, a polygenic response predictor (PRP) was developed to stratify survival benefit from β‐blocker therapy [54]. This PRP distinguished patients who derived substantial survival benefits from β‐blocker exposure from those who did not, suggesting that genetic profiling can guide personalized pharmacological and exercise interventions. Similarly, genetic variants influencing affective responses to physical activity can mediate adherence to exercise programs, thereby impacting training outcomes [55]. For example, certain single nucleotide polymorphisms (SNPs) in the FTO and BDNF genes were associated with more positive affective responses during physical activity, although direct prediction of physical activity levels was not established.

The integration of genetic testing into exercise medicine also faces challenges related to the heterogeneity of exercise protocols and population differences. Systematic reviews of endurance exercise training in animal models demonstrate high variability in training outcomes influenced by factors such as strain, sex, and training duration [56]. Moreover, sex‐specific differences in exercise‐induced adaptations have been observed, with females and males exhibiting distinct physiological and molecular responses to similar training regimens [57]. These biological variabilities necessitate consideration of genetic background and sex in designing personalized exercise prescriptions.

Genetic testing holds promise for predicting individual responses to exercise training by identifying specific gene polymorphisms and polygenic risk scores that influence physiological adaptations. The ACE I/D polymorphism exemplifies how genetic variability can affect exercise outcomes, and genome‐wide approaches are beginning to uncover additional loci relevant to exercise adaptability. Pharmacogenomics complements this by guiding combined exercise and drug therapy optimization. However, the complex interplay of genetics, sex, and environmental factors requires further research to develop robust, clinically applicable predictive models. Ultimately, integrating genetic testing into exercise medicine may enable precision exercise prescriptions tailored to individual genetic profiles, maximizing therapeutic efficacy and health benefits (evidence tier: genetic testing for exercise response—Tier III; see Table 1 for complete summary).

## 5. Population‐Specific CR Strategies

Tailoring rehabilitation to address the distinct needs of various patient subgroups is crucial for optimizing outcomes. The principal considerations and recommended strategies for key populations are summarized in Table 4.

**TABLE 4:** Population‐specific considerations and tailored strategies in cardiac rehabilitation.

Patient population	Key challenges/characteristics	Recommended exercise and management considerations
Elderly cardiovascular patients	• Frailty and sarcopenia: decreased physiological reserve, muscle mass loss. • Multimorbidity and polypharmacy: high prevalence of comorbidities, complex medication regimens increasing fall and interaction risks. • Cognitive impairment: may affect adherence and safety. • Reduced functional capacity and balance: higher risk of falls and fractures.	• Comprehensive geriatric assessment (CGA): Mandatory initial step to evaluate frailty, cognition, and medication burden. • Exercise prescription: prioritize safety, functional independence, and balance training. Consider supervised HIIT cautiously in selected, stable patients. • Multimodal approach: integrate physical therapy, nutritional support, and psychosocial interventions. • Technology use: adapted telemonitoring and wearable devices to enhance supervision and adherence.

Women	• Biological factors: hormonal fluctuations (menstrual, pregnancy history, and menopause) influencing CV risk and exercise response. • Psychosocial barriers: higher burden of depression, anxiety, caregiving duties, and social isolation. • Referral and attendance gaps: less likely to be referred to and complete traditional center‐based CR. • Unique conditions: higher prevalence of conditions like SCAD; breast cancer survivors with cardiotoxicity.	• Holistic, flexible models: Offer home‐based, community‐based, or hybrid CR programs to overcome logistical and social barriers. • Integrated psychological support: essential component to address depression and improve quality of life. • Tailored education and support: consider reproductive history and female‐specific cardiovascular conditions (e.g., SCAD, pregnancy‐related disorders). • Outcome expectations: may see greater improvements in psychosocial outcomes compared to cardiorespiratory fitness gains.

Patients with heart failure (HFrEF/HFpEF)	• Exercise intolerance and fatigue: primary symptom limiting activity. • Skeletal muscle dysfunction and sarcopenia: contributes to fatigue and poor prognosis. • Fear of exercise and low self‐efficacy: common psychological barriers. • Neurohormonal activation: sympathetic overactivity. • Special subgroups: LVAD recipients, heart transplant patients.	• Combined aerobic and resistance training: foundation of exercise therapy. Aerobic training improves VO_2_peak; resistance training counters sarcopenia. • Personalized intensity: guided by cardiopulmonary exercise testing (CPET). Moderate‐intensity often recommended; HIIT may be beneficial for LVAD patients. • Address adherence barriers: utilize behavioral strategies, telehealth support, and address fears directly. • Monitor molecular/functional response: utilize biomarkers (NT‐proBNP, miRNAs) and functional tests to guide progression.

Patients with arrhythmias (e.g., atrial fibrillation (AF))	• Dose–response relationship: moderate exercise reduces AF burden, but prolonged high‐intensity endurance exercise may increase AF risk via atrial remodeling. • Autonomic dysregulation: imbalance can be proarrhythmic. • Safety with devices: patients with pacemakers or ICDs require careful monitoring to prevent inappropriate shocks.	• Preferred modality: moderate‐intensity continuous training (MICT). Balances CV benefits with minimized arrhythmogenic risk. • Autonomic modulation: exercise training aimed at improving sympathovagal balance (↑ HRV). • Vigilant monitoring: essential for patients with implantable devices. Utilize heart rate monitors and symptom diaries. • Innovative delivery: exergames and telerehabilitation with ECG monitoring show promise for engagement and safe remote management.

### 5.1. Special Considerations for Elderly Cardiovascular Patients

Elderly patients with CVD present unique challenges in CR and exercise prescription due to the complex interplay of aging‐related physiological changes, comorbidities, polypharmacy, and frailty. A comprehensive assessment that includes evaluation of frailty status, cognitive function, and medication burden is essential to tailor interventions appropriately and minimize risks such as orthostatic hypotension, falls, and adverse drug interactions. Frailty, a multidimensional syndrome characterized by decreased physiological reserve and increased vulnerability to stressors, is highly prevalent in older adults with CVD and strongly predicts poorer outcomes including exercise intolerance and increased morbidity. Incorporating frailty assessment tools into clinical practice allows for stratification of patients and guides individualized rehabilitation strategies. Cognitive impairment, common in this population, further complicates adherence and safety during exercise programs, necessitating close monitoring and potentially involving caregivers in the rehabilitation process. Polypharmacy, often present due to multiple comorbidities, increases the risk of drug–drug interactions and adverse events, especially when antihypertensive agents are used, requiring careful medication review and adjustment. Recent evidence underscores the safety and efficacy of high‐intensity interval training (HIIT) in selected elderly patients, demonstrating improvements in cardiorespiratory fitness and functional capacity without increased adverse events. However, implementation should be cautious and individualized, considering the patient’s overall health status and functional ability. Emphasis on balance training and functional exercises is critical to prevent falls and fractures, which are major causes of disability and mortality in this age group. Multidisciplinary approaches integrating physical therapy, nutritional support, and psychosocial interventions have shown promise in improving outcomes. Technological advances such as telemonitoring and wearable devices offer opportunities to enhance supervision and adherence, although usability must be adapted to the elderly population’s needs. Overall, the integration of comprehensive geriatric assessment with personalized exercise prescriptions that prioritize safety, functional independence, and quality of life represents the future direction of CR in elderly cardiovascular patients [58–65].

### 5.2. Clinical Practice Recommendations for Elderly CR Patients

Perform comprehensive geriatric assessments (including frailty screening using tools such as the fried frailty index, cognitive assessment, and medication reconciliation) at CR entry for all patients aged ≥ 75 years or with suspected frailty. Initiate exercise at low‐to‐moderate intensity (e.g., 40%–60% of heart rate reserve) and progress cautiously based on symptom response, functional assessment, and daily monitoring. Incorporate balance training and functional exercises at least twice weekly to reduce fall risk; include resistance training to combat sarcopenia. Use wearable devices with simplified interfaces and involve caregivers in monitoring and encouragement; adapt technology for age‐related visual and dexterity limitations. Consider HIIT only in selected stable patients after thorough functional assessment and under direct supervision for initial sessions; prefer MICT for most elderly patients. Integrate multidisciplinary care involving physical therapy, nutritional support, and psychosocial interventions; regularly review medications to minimize orthostatic hypotension and drug interactions.

### 5.3. Characteristics of CR in Women

CR tailored to women presents unique challenges and considerations that stem from biological, psychosocial, and disease‐specific factors. A critical aspect influencing women’s participation and outcomes in CR is the fluctuating hormonal milieu, which encompasses changes related to menstrual cycles, pregnancy history, and menopausal status. These hormonal variations can affect cardiovascular risk profiles, exercise tolerance, and psychosocial well‐being, necessitating personalized rehabilitation approaches that account for these physiological dynamics. For instance, postmenopausal women often exhibit increased cardiovascular risk due to estrogen deficiency, which may influence exercise prescription and risk factor management during CR. Additionally, pregnancy history, including complications such as preeclampsia or gestational diabetes, imparts long‐term cardiovascular implications that should be integrated into rehabilitation planning.

Beyond biological factors, social and psychological determinants play a more pronounced role in women’s CR engagement and success. Women frequently encounter higher psychosocial barriers, including caregiving responsibilities, social isolation, and depression, which can impede adherence to exercise and lifestyle modifications. Studies have demonstrated that women in CR programs report more severe depressive symptoms and lower quality of life compared to men, factors that correlate with poorer long‐term outcomes and increased mortality risk despite rehabilitation efforts [66]. These findings underscore the necessity of incorporating psychological support and social facilitation strategies within CR programs for women. Community‐ and home‐based physical activity interventions have shown promise in overcoming traditional barriers by offering flexible scheduling, appealing activity modes (e.g., dancing and group walking), and fostering social interaction, thereby enhancing participation and physical activity levels among women [67]. Moreover, women’s completion rates in CR are influenced by social support and insurance status, with family support and Medicare insurance being associated with higher program completion [68].

Special populations of women, such as breast cancer survivors and those with spontaneous coronary artery dissection (SCAD), have distinct CR needs that are increasingly recognized. Breast cancer survivors often face cardiotoxicity from cancer treatments, necessitating tailored CR programs that address both oncologic and cardiovascular rehabilitation goals. Similarly, SCAD predominantly affects younger women and requires specialized secondary prevention strategies, including CR programs sensitive to the unique pathophysiology and psychosocial context of this condition [69]. These subgroups highlight the expanding scope of female‐specific CR, emphasizing the importance of individualized care models that consider reproductive history, cancer survivorship, and rare female‐predominant cardiovascular conditions.

Furthermore, sex differences extend to program delivery and outcomes. Women are less likely to be referred to and attend traditional center‐based CR programs, often due to logistical challenges and perceived lack of support. Hybrid and home‐based CR models have attracted higher proportions of female participants, though completion rates may be lower compared to center‐based programs, indicating a need for programmatic adjustments to enhance adherence [70]. Psychological interventions integrated into CR have demonstrated benefits in reducing depressive symptoms and improving quality of life among elderly women, suggesting that gender‐specific psychological support is a vital component of comprehensive rehabilitation [71]. Additionally, women tend to have smaller improvements in cardiorespiratory fitness but greater gains in psychosocial outcomes following intensive CR programs, highlighting the importance of multidimensional outcome assessment [72].

The characteristics of CR in women are shaped by complex interactions between hormonal status, psychosocial factors, and unique clinical conditions. Effective CR for women requires a holistic, flexible, and patient‐centered approach that addresses biological variability, social determinants, and specific disease contexts. Incorporating community‐based options, psychological support, and tailored interventions for special populations can enhance participation, adherence, and outcomes in female cardiac patients. Ongoing research and clinical innovation are essential to optimize CR delivery and efficacy for women, ultimately reducing sex disparities in cardiovascular health and rehabilitation success.

### 5.4. Clinical Practice Recommendations for Women in CR

Offer flexible delivery options including home‐based, community‐based, or hybrid CR programs to address logistical and caregiving barriers; consider group‐based activities (e.g., walking groups and dance‐based exercises) that appeal to women’s preferences. Integrate routine psychological screening and support, particularly for depression and anxiety, which are more prevalent and influential in women; consider cognitive‐behavioral therapy or mindfulness‐based interventions. Inquire about reproductive history (pregnancy complications such as preeclampsia or gestational diabetes, menopausal status, and hormone therapy) and female‐predominant conditions (SCAD and breast cancer history) during initial assessment. Recognize that women may show smaller cardiorespiratory fitness improvements but meaningful psychosocial gains; assess and celebrate multidimensional outcomes beyond VO_2_peak alone. Address referral disparities through proactive outreach and patient education about CR benefits; engage family members to support participation.

## 6. Exercise Rehabilitation for Special CVD Populations

### 6.1. Exercise Management in Patients With HF

Exercise management in patients with HF has evolved significantly, with recent guidelines emphasizing individualized aerobic and resistance combined training programs to optimize patient outcomes. Aerobic exercise, often prescribed based on CPET, remains a cornerstone of CR, improving VO_2_peak, exercise tolerance, and quality of life across various HF phenotypes, including HF with reduced ejection fraction (HFrEF) and preserved ejection fraction (HFpEF) [73, 74]. Moderate‐intensity exercise training, sometimes combined with resistance or inspiratory muscle training, has demonstrated superiority in improving functional capacity and cardiac diastolic function compared to high‐intensity protocols in certain HF populations [75, 76]. The integration of resistance training addresses skeletal muscle weakness and sarcopenia, common in HF patients, further enhancing exercise capacity and quality of life [77].

In patients with advanced HF supported by left ventricular assist devices (LVADs) or those who have undergone heart transplantation, exercise prescription requires special considerations. Exercise training in LVAD recipients has been shown to be safe and effective, with HIIT improving cardiorespiratory fitness and exercise tolerance without significant adverse events [78]. Similarly, heart transplant recipients exhibit persistent exercise intolerance due to cardiac denervation and peripheral abnormalities; however, tailored aerobic and strengthening exercise interventions, including HIIT, can partially restore exercise capacity and improve quality of life [79]. These patients require careful risk stratification and individualized exercise protocols, often guided by CPET parameters, to maximize benefits while minimizing risks [80].

Mechanistically, exercise training improves myocardial energy metabolism and reduces maladaptive remodeling in HF. Studies have highlighted the role of exercise in enhancing mitochondrial function, optimizing substrate utilization, and modulating key signaling pathways implicated in myocardial fibrosis and inflammation, such as the MAPK and ubiquitin‐proteasome systems [81–83]. Exercise‐induced improvements in miRNA profiles, including increased plasma levels of miR‐126, correlate with enhanced cardiac function and exercise tolerance in HFpEF patients, suggesting molecular adaptations underpinning clinical benefits [84]. Furthermore, exercise mitigates sympathetic overactivation and neurohormonal dysregulation, common in HF pathophysiology, contributing to improved hemodynamics and reduced disease progression [85, 86].

Adherence to exercise regimens remains a significant challenge in HF management. Psychological factors, such as negative attitudes, low self‐efficacy, and fear of exercise, alongside social support deficits, are key barriers to sustained participation in home‐based or outpatient CR programs [87, 88]. Interventions targeting these mediators, including behavioral strategies and telehealth modalities, have shown promise in improving long‐term adherence and functional outcomes [89]. The development of knowledge‐based systems for personalized exercise prescription, integrating patient‐specific clinical and functional data, represents a future direction to optimize exercise therapy in HF [90].

The latest guidelines advocate for a personalized, combined aerobic and resistance training approach in HF patients, tailored to individual functional capacity and comorbidities. Special populations such as LVAD recipients and heart transplant patients require modified exercise protocols with close monitoring. Exercise training exerts beneficial effects on myocardial energy metabolism, neurohormonal balance, and molecular pathways involved in HF progression. Addressing adherence barriers through targeted interventions and leveraging technology will be critical in maximizing the therapeutic potential of exercise in this population.

### 6.2. Clinical Practice Recommendations for HF Patients

Prescribe combined aerobic and resistance training as first‐line exercise therapy, with aerobic training at moderate intensity (e.g., 60%–70% of heart rate reserve, or 11–13 on Borg RPE scale) guided by CPET when available. For patients with LVADs or post‐heart transplantation, initiate exercise under direct supervision with careful monitoring of hemodynamic responses, device parameters, and symptoms; progress gradually over 4–6 weeks before considering independent home exercise. Monitor NT‐proBNP and functional capacity (VO_2_peak and 6MWT) at baseline and periodically (every 4–6 weeks) to track response and adjust exercise prescriptions; discuss biomarker trends with patients to enhance motivation. Address adherence barriers through behavioral counseling, telehealth support, shared decision‐making about exercise goals, and involvement of family members. For patients with significant anxiety or fear of exercise, consider graded exposure starting with low‐intensity seated exercises before progressing to standing and walking activities.

### 6.3. Exercise Strategies for Patients With Arrhythmias

Exercise management in patients with arrhythmias, particularly atrial fibrillation (AF) and those with implanted cardiac devices, requires a nuanced understanding of the interplay between physical activity intensity, arrhythmia risk, and autonomic nervous system modulation. The dose‐response relationship between exercise intensity and AF recurrence risk is a critical consideration. Moderate‐intensity continuous training (MICT) has been shown to reduce AF incidence and symptom burden, making it the preferred exercise modality for AF patients. Conversely, prolonged high‐intensity endurance exercise may increase AF risk due to mechanisms such as irreversible atrial remodeling, heightened inflammation, and increased vagal tone, which predispose to arrhythmogenesis [91]. This highlights the importance of tailoring exercise prescriptions to balance cardiovascular benefits with arrhythmia risk.

For patients with implantable cardiac devices such as pacemakers or implantable cardioverter defibrillators (ICDs), exercise safety is paramount. Case reports and clinical observations indicate that carefully monitored, stepwise exercise programs incorporating heart rate monitoring and medication adjustments can enable safe resumption of physical activity even after exercise‐induced ICD shocks [92]. Hybrid comprehensive telerehabilitation with ECG telemonitoring in HF patients, many of whom have arrhythmias, demonstrated safety without symptomatic arrhythmias necessitating program discontinuation, underscoring the feasibility of remote monitoring during rehabilitation [93]. These findings advocate for individualized exercise regimens with vigilant monitoring in device patients to mitigate arrhythmic risks.

Exercise training also exerts antiarrhythmic effects through modulation of autonomic nervous system balance. Studies in animal models of spinal cord injury revealed that exercise training blunted sympathetic stimulation‐induced arrhythmia risk, suggesting that exercise can restore sympathovagal equilibrium and reduce arrhythmogenic susceptibility [94]. In humans, sympathovagal balance assessed via HRV during deep sleep predicted arrhythmia occurrence after high‐volume endurance exercise, indicating that autonomic regulation plays a pivotal role in exercise‐induced arrhythmogenesis [95]. This autonomic modulation likely underpins the protective effects of moderate exercise against arrhythmias.

In AF management, CR incorporating tailored exercise improves exercise capacity and quality of life, and it reduces AF burden. Chinese expert consensus emphasizes multidimensional rehabilitation evaluation and individualized exercise prescriptions for AF patients, reflecting the growing recognition of exercise as a cornerstone of AF management [96]. Emerging technologies such as exergames offer promising remote, engaging platforms for exercise delivery in AF, potentially enhancing adherence and outcomes, though further validation is needed [97].

In summary, exercise strategies for arrhythmia patients should prioritize moderate‐intensity training to minimize AF recurrence risk while harnessing autonomic modulation benefits. For patients with implantable devices, personalized, closely monitored exercise programs are safe and effective. Understanding the dose–response relationship and autonomic mechanisms is essential for optimizing exercise prescriptions to improve cardiovascular health without exacerbating arrhythmic risk.

### 6.4. Clinical Practice Recommendations for Arrhythmia Patients

Recommend moderate‐intensity continuous training (MICT) as the preferred exercise modality for AF patients to optimize cardiovascular benefits while minimizing arrhythmia risk; target 40%–60% of heart rate reserve. For patients with implantable cardiac devices, develop individualized exercise protocols with heart rate monitoring, symptom diaries, and medication adjustment; involve cardiology/electrophysiology teams in exercise prescription. Consider hybrid telerehabilitation with ECG telemonitoring for enhanced safety and early detection of arrhythmic events during exercise; advise patients to avoid exercise during active arrhythmia symptoms. Educate patients about the dose–response relationship: moderate exercise is protective (reduces AF burden by ∼30–40%); prolonged high‐intensity endurance exercise (> 10 h/week of vigorous activity) may paradoxically increase AF risk through atrial remodeling. For patients with recent ICD shocks, implement stepwise exercise programs starting with low‐intensity walking (10–15 min) and progressing by 5‐min increments weekly, with close monitoring of symptoms and device interrogation at each clinic visit.

## 7. Future Development Directions of CR Exercise Medicine

The future trajectory of CR is being shaped by several converging technological and scientific advancements. Table 5 outlines the potential applications and challenges of key emerging technologies poised to transform the field. It is important to note that the technologies discussed in this section—particularly VR/XR, blockchain, digital twins, and advanced omics—are predominantly at Tier IV (speculative) evidence level, with limited or no CR‐specific clinical data. They are presented to illustrate potential future directions rather than current clinical recommendations.

**TABLE 5:** Emerging technologies and their potential roles in future cardiac rehabilitation.

Technology	Core function/principle	Potential application in cardiac rehabilitation	Key challenges/considerations
Virtual reality (VR) and extended reality (XR)	Creates immersive, interactive simulated environments. Operates via distraction therapy, reducing perception of effort, pain, and anxiety.	• Enhancing engagement and adherence: makes repetitive exercises enjoyable and motivating. • Pain and anxiety management: particularly useful post‐cardiac surgery or during stressful phases. • Safe environment for training: Allows practice of daily activities or exposure to controlled stress scenarios in a risk‐free setting.	• Content development: need for clinically validated, CR‐specific exercise and education modules. • User Accessibility & Acceptance: Concerns regarding cost, technological literacy, motion sickness, and accessibility for elderly patients. • Integration into workflow: defining how and when to use VR within a comprehensive CR program.

Artificial intelligence and machine learning (AI/ML) (existing applications with expanding future potential)	Algorithms that learn patterns from large datasets to predict, classify, and optimize. Includes computer vision (CV) for movement analysis and natural language processing (NLP) for understanding text/speech.	• Precision exercise prescription: AI models analyze multimodal data (biomarkers, genetics, wearables) to generate and dynamically adjust personalized exercise plans. • Risk prediction and early warning: identifies patients at high risk of dropout or adverse events. • Automated monitoring and feedback: CV provides real‐time posture correction; NLP offers automated coaching and analyzes patient sentiment.	• Data quality and quantity: requires large, high‐quality, and labeled datasets for training robust models. • Algorithmic transparency and explainability: “black box” problem–need for interpretable AI to gain clinician trust. • Clinical integration and validation: must complement, not replace, clinical judgment. Requires rigorous RCTs to prove efficacy.

Digital twin	A dynamic virtual model of an individual patient, built using their anatomical, physiological, and genetic data. It simulates responses to interventions in real‐time.	• Personalized simulation and optimization: “test” different rehabilitation protocols on the digital twin to predict the optimal one for the actual patient before implementation. • Predictive complication Management: forecasts potential adverse responses (e.g., arrhythmia, hypotension) to exercise adjustments. • Patient education and engagement: visualizing one’s own “digital twin” can improve understanding and motivation.	• Data intensity and modeling complexity: requires vast amounts of personal data and sophisticated multiscale physiological models. • Validation and accuracy: difficult and expensive to validate the predictive fidelity of a digital twin. • Ethical and privacy concerns: Ownership and use of highly detailed personal health avatars.

Blockchain	A decentralized, distributed digital ledger that records transactions immutably and transparently.	• Secure data exchange and interoperability: enables safe sharing of patient data from wearables, apps, and EHRs among the multidisciplinary CR team. • Patient data sovereignty and consent management: gives patients control over who accesses their data and for what purpose. • Integrity of remote monitoring data: ensures data from home‐based CR is tamper‐proof, enhancing trust in remote decisions.	• Technical complexity and scalability: integration with existing healthcare IT systems is nontrivial. • Regulatory and legal frameworks: healthcare regulations (like HIPAA) need to align with blockchain’s decentralized nature. • Energy consumption and speed: some blockchain implementations have high computational costs.

Advanced omics and microbiome analysis	High‐throughput molecular profiling (genomics, single‐cell sequencing, metagenomics) to understand individual biological variability at a deep level	• Ultra‐personalized prescription: integrating genetic risk scores, single‐cell response profiles, and gut microbiome composition to tailor exercise, diet, and adjunct therapies. • Novel biomarker discovery: identifying new molecular signatures of exercise response and cardiovascular resilience. • Mechanistic insights: uncovering new pathways linking exercise, gut health, and cardiovascular outcomes for targeted interventions.	• Cost and accessibility: currently expensive and not routinely available, risking healthcare disparities. • Clinical interpretation: translating complex omics data into actionable clinical guidelines is challenging. • Longitudinal data needs: requires repeated measurements to understand dynamic changes, not just a single snapshot.

### 7.1. Multimodal Technology Integration Platforms

VR creates immersive exercise environments that enhance engagement via distraction therapy, reducing pain and anxiety. VR has shown efficacy in postoperative pain reduction and improved physiological parameters in cardiac surgery patients, though CR‐specific evidence is limited to small feasibility studies [98].

Blockchain secures medical data integrity and enables transparent sharing across multidisciplinary teams via decentralized ledgers, supporting remote cardiac telerehabilitation [99].

Digital twins create virtual patient replicas to simulate and optimize rehabilitation protocols, predicting cardiovascular responses and potential complications [100].

Collectively, VR, blockchain, and digital twins form a synergistic multimodal platform supporting home‐based and hybrid rehabilitation. Preliminary studies show comparable outcomes to center‐based programs [101–103]. However, challenges remain: high‐quality RCTs, user literacy, equitable access, and patient‐centered design are needed.

CR‐specific evidence for VR is limited; large RCTs and long‐term follow‐up are urgently needed. User acceptability among elderly patients remains uncertain, with motion sickness and technical complexity as potential barriers (evidence tier: VR/XR—Tier IV; blockchain—Tier IV; digital twins—Tier IV; see Table 1 for complete summary).

### 7.2. In‐Depth Applications of Precision Medicine

The following approaches are emerging frontiers not yet ready for routine clinical application.

Single‐cell sequencing enables granular analysis of molecular responses to exercise, revealing cellular heterogeneity in cardiac and skeletal muscle adaptation and identifying novel biomarkers [104].

The gut microbiome–exercise–cardiovascular axis influences metabolism, immunity, and inflammation. Microbiome profiling combined with AI may enable personalized dietary and probiotic strategies to enhance cardiovascular rehabilitation [105].

Epigenetic clocks based on DNA methylation patterns assess biological aging and exercise impact, potentially guiding individualized rehabilitation [106].

These advances are shifting CR toward precision medicine. Integration with digital health technologies (wearables and telemedicine) enables real‐time monitoring and adaptive rehabilitation management [25]. As these technologies mature, they may transform CR into a highly tailored discipline (evidence tier: advanced omics/microbiome—Tier IV; see Table 1 for complete summary).

### 7.3. Implementation Science: Overcoming Barriers to Clinical Translation

Translation of technology‐enabled CR faces multilevel barriers: Patient‐level: low digital literacy, limited access, low self‐efficacy, fear of technology, and privacy concerns. Clinician‐level: limited training, skepticism, workflow disruption, and lack of time. Health system‐level: reimbursement gaps, interoperability issues, fragmented data, and insufficient staffing. Policy‐level: evolving regulations, privacy concerns, and lack of standardization. A comprehensive summary of these multilevel barriers, together with recommended implementation strategies and supporting references, is provided in Table 6.

**TABLE 6:** Implementation barriers and strategies for technology‐integrated cardiac rehabilitation.

Barrier domain	Specific barriers	Recommended implementation strategies	Key evidence/references
Patient‐level	Low digital literacy; limited technology access/ownership; health literacy deficits; low self‐efficacy and motivation; fear of technology; concerns about data privacy	Simplified user interfaces; hands‐on training sessions; peer support programs; community partnerships for device access; culturally tailored education materials; patient navigators	Hirai et al. 2025 [41]; Ghisi et al. 2023 [43]; Bola et al. 2026 [42]
Clinician‐level	Limited training in digital health/AI; skepticism about technology; workflow disruption concerns; lack of time for remote monitoring; uncertainty about AI recommendations	Dedicated training programs; clinical champions and early adopters; user‐centered design of interfaces; integration with existing workflows; clear protocols for alert management; shared decision‐making tools	Rigamonti et al. 2020 [46]; Hirai et al. 2025 [41]; Su et al. 2025 [36]
Health System‐level	Reimbursement gaps; interoperability issues; fragmented data systems; insufficient staffing; technology procurement challenges; rapid technology evolution	Policy advocacy for reimbursement models; standardized interoperability protocols; hybrid care models; dedicated implementation teams; phased technology rollout; ongoing funding models	Falter et al. 2020 [99]; Bhatla et al. 2024 [30]; Antoniou et al. 2022 [31]
Technology‐level	Regulatory uncertainty; validation gaps; accuracy limitations; lack of standardization; platform fragmentation; cybersecurity concerns	Regulatory guidance and certification pathways; rigorous validation studies; standards development; quality assurance protocols; security audits; transparent algorithms	Su et al. 2025 [36]; Kitagaki et al. 2025 [39]; Hirai et al. 2024 [40]
Policy‐level	Fragmented regulations; privacy concerns; equity gaps; lack of national standards; reimbursement uncertainty	Legislative alignment across jurisdictions; robust privacy frameworks and consent management; equity‐focused policies; national/regional standards development; stakeholder engagement in policy making	Lam et al. 2022 [103]; Bhatla et al. 2024 [30]; Falter et al. 2020 [99]

Implementation science frameworks (CFIR and RE‐AIM) offer structured approaches to identify barriers and facilitators. CFIR categorizes determinants across five domains; RE‐AIM evaluates reach, effectiveness, adoption, implementation, and maintenance.

Strategies to promote implementation include clinician training with clinical champions; user‐centered design involving patients and clinicians; hybrid care models combining remote and in‐person care; health equity‐focused policies addressing access disparities; and sustainable financing models.

Integrating implementation science into CR research is essential for translating innovations into sustainable practice improvements.

## 8. Limitations

This review has several limitations that should be acknowledged. First, as a narrative review, it is subject to potential selection bias in the literature cited, and the broad scope necessarily limits the depth of coverage for any single domain. Second, many of the technologies and approaches discussed—particularly digital twin modeling, blockchain applications, single‐cell omics, and microbiome‐based interventions—are supported predominantly by early‐phase studies, proof‐of‐concept investigations, or protocol papers rather than large‐scale randomized controlled trials. The evidence base for AI‐driven exercise prescription and biomarker‐guided protocols, while promising, remains largely at the developmental or validation stage, and clinical translation has not yet been widely achieved. Third, the integration of precision medicine into routine CR faces significant practical barriers—including cost, accessibility, standardization of assays, and clinical interpretability—that are not yet fully resolved. Fourth, while we discuss population‐specific strategies, the literature on ethnic minorities and underserved populations remains sparse, limiting the generalizability of our recommendations. Fifth, the rapid pace of technological evolution means that some findings may become outdated quickly, and long‐term outcome data for many digital health interventions are still lacking. Finally, the heterogeneity of study designs, patient populations, and rehabilitation protocols across the cited literature precludes quantitative synthesis. These limitations underscore the need for continued rigorous research, including large randomized controlled trials, implementation science studies, and health economic evaluations, to translate these promising innovations into equitable and sustainable clinical practice.

## 9. Conclusion

The evolution of CR is being reshaped by technological integration, precision medicine, and population‐specific strategies. These three pillars collectively drive a paradigm shift toward enhanced accessibility and efficacy.

However, enthusiasm must be tempered with critical appraisal of current evidence. Much evidence supporting precision medicine and emerging technologies remains preliminary—from proof‐of‐concept studies, protocols, or small feasibility trials. Clinical translation faces formidable challenges: rigorous RCT validation, protocol standardization, cost‐effectiveness evaluation, and sustainable reimbursement models. Technology access, digital literacy, and infrastructure disparities threaten to widen health inequities if unaddressed.

Realizing CR’s potential requires sustained collaborative efforts among researchers, clinicians, developers, and policymakers. Future research must prioritize large‐scale pragmatic trials and implementation science to identify and overcome adoption barriers.

No single approach suffices. The future of CR lies in the synergistic application of technology, precision medicine, and individualized care, guided by rigorous evidence and ethical considerations. This integrated approach promises to transform CR into a more precise, accessible, and effective discipline improving outcomes for all patients.

**Nomenclature**
ACS: Acute coronary syndrome
AF: Atrial fibrillation
AI: Artificial intelligence
CABG: Coronary artery bypass grafting
CAD: Coronary artery disease
CDSS: Clinical decision support system
CPET: Cardiopulmonary exercise testing
CR: Cardiac rehabilitation.
CVD: Cardiovascular disease
ECG: Electrocardiogram
GWAS: Genome‐wide association study
HF: Heart failure
HFpEF: Heart failure with preserved ejection fraction
HFrEF: Heart failure with reduced ejection fraction
HIIT: High‐intensity interval training
HIAIT: High‐intensity aerobic interval training
HRV: Heart rate variability
HRVB: Heart rate variability biofeedback
hs‐CRP: High‐sensitivity C‐reactive protein
ICD: Implantable cardioverter defibrillator
IoT: Internet of things
ISWT: Incremental shuttle walk test
LVAD: Left ventricular assist device
mHealth: Mobile health
MI: Myocardial infarction
MICT: Moderate‐intensity continuous training
miRNA: microRNA
ML: Machine learning
NLP: Natural language processing
NT‐proBNP: N‐terminal pro‐B‐type natriuretic peptide
PPS: Polygenic predictor score
PRP: Polygenic response predictor
SCAD: Spontaneous coronary artery dissection
SNP: Single nucleotide polymorphism
SpO_2_
: Blood oxygen saturation
TAVR: Transcatheter aortic valve replacement
VEGF: Vascular endothelial growth factor
VO_2_peak: Peak oxygen uptake
VR: Virtual reality
XR: Extended reality

## Author Contributions

Li Wei conceived the study concept and design, supervised the project, and acquired funding. Tong Liu and Yuting Xia performed the literature search, data collection, and data analysis. Tong Liu wrote the original draft. Yuting Xia and Li Wei critically revised the manuscript for important intellectual content.

## Funding

This study was supported by the Medical Research Project Plan of Sichuan Province (S22068).

## Disclosure

All authors read and approved the final manuscript and agreed to be accountable for all aspects of the work.

## Ethics Statement

The authors have nothing to report.

## Consent

The authors have nothing to report.

## Conflicts of Interest

The authors declare no conflicts of interest.

## Data Availability

The data used to support the findings of this study are included within the article.
